# *In Silico* Prediction and *In Vivo* Validation of *Daphnia pulex* Micrornas

**DOI:** 10.1371/journal.pone.0083708

**Published:** 2014-01-06

**Authors:** Shuai Chen, Garrett J. McKinney, Krista M. Nichols, Maria S. Sepúlveda

**Affiliations:** 1 Department of Forestry and Natural Resources, Purdue University, West Lafayette, Indiana, United States of America; 2 Department of Biological Sciences, Purdue University, West Lafayette, Indiana, United States of America; 3 Northwest Fisheries Science Center, National Ocean and Atmospheric Administration, Seattle, Washington State, United States of America; Rutgers - New Jersey Medical School, United States of America

## Abstract

*Daphnia pulex*, the crustacean with the first sequenced genome, is an important organism that has been widely used in ecological and toxicological research. MicroRNAs (miRNAs) are 21–25 nucleotide small non-coding RNAs that are involved in a myriad of physiological processes. In this research, we predicted 75 *D. pulex* miRNAs by sequence homology and secondary structure identification from the full genome sequence. Fourteen predicted miRNAs were selected for quantitative real time polymerase chain reaction (RT-PCR) validation. Out of these, eight (mir-8, mir-9, mir-12, mir-92, mir-100, mir-133, mir-153 and mir-283) were successfully amplified and validated. Next, expression levels were quantified at three different life stages (days 4, 8 and 12 of age) using U6 spliceosomal RNA as a reference gene. The expression of mir-8, mir-9, mir-12, mir-92 and mir-100 significantly differed across time suggesting these microRNAs might play a critical role during *D. pulex* development. This is the first study to identify and validate miRNAs in *D. pulex*, which is an important first step in further studies that evaluate their roles in development and response to environmental and ecological stimuli.

## Introduction

*Daphnia pulex* is an ecologically important organism in freshwater ecosystems that has been studied for decades [1] and is the first crustacean for which a full genome sequence is available. *D. pulex* has a short life cycle and the phenotypic plasticity displayed by these organisms makes it an ideal model species for ecological, toxicological, and evolutionary research [2]. *D. pulex* can reproduce by either clonal (parthenogenetic) or sexual reproduction and often undergoes cyclical parthenogenesis. Usually, a healthy population will be genetically identical females that produce diploid parthenogenetic eggs. Haploid sexual eggs can be produced when females encounter certain environmental cues such as starvation or highly crowed conditions [3], [4]. Diploid juvenile *D.pulex* usually take 6–10 days and go through 5 or 6 juvenile instars to reach sexual maturity [4]. After reaching maturity, the growth rate of *D. pulex* decreases dramatically as energy is focused mostly on breeding rather than somatic development [4], [5]. Interestingly, *D. pulex* populations that are genetically identical can still show plasticity in certain phenotypes, e.g. predator induced helmet and “neckteeth” formation [6]. Thus clonal lines with phenotypically differential individuals enable us to examine whether there is an epigenetic influence on phenotype [7]. All these unique biological attributes, paired with the newly sequenced *D. pulex* genome, provide an unparalleled opportunity to study the epigenetic signatures in *D. pulex* and to examine how these signatures are changed and/or inherited across environmental conditions.

Micro-RNAs (miRNAs) are short, non-coding endogenous RNAs that are approximately 20–25 nucleotides in length. Recent research has revealed that miRNAs are involved in a variety of aspects of animal development including muscle development, aging and body size regulation [8]–[10]. During miRNA biogenesis, miRNA genes need to form a hairpin loop of ∼70 nucleotide in length [11]. The conservation of miRNA sequences across taxa, together with the secondary hairpin structure provide an opportunity to predict conserved miRNAs in species for which miRNAs have not been previously described. However, *in silico* prediction does not necessarily mean those predicted miRNAs exist functionally in an organism. Therefore, *in vivo* validation of predicted miRNAs is needed. Several approaches are available for this validation including real-time polymerase chain reaction (RT-PCR) and cloning and deep sequencing.

According to miRBase (Release 19), one of the largest miRNA databases [12], no *D. pulex* miRNAs have been experimentally verified. The objectives of this research were to 1) predict conserved *D*. *pulex* miRNAs by sequence homology and hairpin structure identification; 2) further validate predicted miRNAs by end point PCR and RT-PCR; 3) select stable reference genes for *D. pulex* miRNA expression; and 4) examine the expression level of validated miRNAs during *D. pulex* development. This research provides a foundation for future research on the role of *D. pulex* miRNAs during development.

## Materials and Methods

### *In silico* prediction of *D*. *pulex* miRNAs

Candidate miRNA loci were identified by conducting a nucleotide BLAST (BLAST-2.2.25, e-value 0.1)[13] using all animal mature miRNA sequences available (miRBase Release 19 http://www.mirbase.org/) against the *D*. *pulex* genome (DOE Joint Genome Institute http://www.jgi.doe.gov/). Only miRNA loci with at least 18 nucleotides with no more than 2 mismatches were considered for further analyses. In addition, if several miRNAs from the same miRNA family (e.g. has-mir-9 and mmu-mir-9 were considered from mir-9 family) matched the same *D. pulex* locus, the miRNA sequence with the highest identity was chosen.

### Pre-miRNA hairpin structure identification

During animal miRNA biogenesis, miRNA genes are initially transcribed into long primary miRNAs (pri-miRNAs) and then processed into ∼70 nucleotide precursor miRNAs (pre-miRNAs) that later form hairpin structures [11]. Identification of these pre-miRNA hairpin structures plays a critical role in miRNA computational prediction. We used a custom Perl script (File S1) to obtain the 200 nucleotide flanking sequence which center surrounded the candidate *D. pulex* miRNA loci (identified from BLAST above) as potential pre-miRNAs. These potential pre-miRNAs were then analyzed by Mfold [14] using default settings. For each potential pre-miRNA, Mfold outputs several structures with different minimum folding energy (MFE). We inspected structures that had the lowest MFE and only those that fulfilled the following criteria were considered to be authentic hairpin structures: 1) pre-miRNAs could form an appropriate hairpin structure with the potential mature miRNA located on one of the hairpin arms; 2) less than six mismatches were observed between the potential mature miRNA and its opposite strand; and 3) no breaks occurred between the mature miRNA and its opposite strand.

### *In vivo* validation of *D*. *pulex* miRNAs

#### Animal culture and sample preparation

*D. pulex* were obtained from Dr. John Colbourne's lab at Indiana University (Bloomington, IN, USA). Organisms were cultured in hard water (NaHCO_3_ 0.192 g/L, CaSO_4_·2H_2_O 0.120 g/L, MgSO_4_ 0.120 g/L, KCl 0.008 g/L) and maintained in an environmental chamber at 25°C on a 16/8 light/dark cycle. Water was changed twice a week and *D*. *pulex* were fed YCT (yeast, cereal leaf, trout chow) mix (Aquatic Research Organisms Inc., Hampton, NH, USA) after every water change. *D*. *pulex* were collected from a single brood at 4, 8 and 12 days of age. At each time point, 3 samples of 10 individuals each were collected and flash frozen in liquid nitrogen. miRNAs (and small RNAs) were extracted using PureLink miRNA isolation kit (Invitrogen, Carlsbad, CA, USA). Total miRNA was quantified using a Qubit Fluorometer (Invitrogen, Carlsbad, CA, USA) and miRNA extracts were stored at −80°C until PCR analysis.

#### End-point PCR and RT-PCR validation

A subset of predicted miRNAs (n = 14) were randomly chosen for validation. Specific primers (Table S2) were designed following the method described by Chen *et al*. (2005) [15]. The protocol for miRNA validation was adapted from Varkonyi-Gasic *et al*. (2007) [16]. Briefly, miRNAs were first reverse-transcribed using 2 pmol miRNA specific stem-loop primers, a mix of 0.5 mM dNTP, and 1 µg of RNA template heated at 65°C for 5 min. A 1X first-strand buffer containing 5 mM DTT, 2 units of RNase OUT and 2.5 units of SuperScript III were then added and the mix incubated on ice for another 2 min. The final mix was incubated at 16°C for 30 min followed by 60 cycles at 30°C for 30 s, 42°C for 30 s and 50°C for 1 s and then incubated at 85°C for 5 min to inactivate the reverse transcriptase. cDNA was quantitated using a Qubit Fluorometer and stored at −80°C. The subsequent end-point PCRs were performed by mixing 0.5 mM dNTP, 0.2 µM forward primer, 0.2 µM universal reverse primer, 1 unit of Advantage 2 Polymerase mix (Clontech, Mountain View, CA, USA), 1 µl cDNA and nuclease-free water into a 20 µl volume reaction. PCR conditions were 94°C for 2 min, followed by 30–40 cycles at 94°C for 15 s and 60°C for 1 min. PCR products were visualized by electrophoresis on a 4% agarose gel.

RT-PCR was conducted on a StepOnePlus real-time PCR system (Applied Biosystems, Inc. Foster City, CA, USA). Each reaction contained 2 µM SYBR Green I master mix, 1 µM forward primer, 1 µM reverse primer and 2 µl RT product. Cycling parameters were 95°C for 5 min, followed by 35–45 cycles at 95°C for 5 s, 60°C for 10 s and 72°C for 1 s. A melting curve analysis was performed to check that no primer-dimers were present.

#### Expression of *D.pulex* miRNAs at different life stages

To better understand the role of miRNAs in *D. pulex* development, we further tested the expression of a validated set of miRNAs at different ages. Several widely used small RNA reference genes in other model species were selected as candidate reference genes. A nucleotide BLAST (BLAST-2.2.25, e-value 0.1) search was conducted using candidate reference gene sequences against the *D. pulex* genome. The BLAST hits on *D. pulex* genome were used for primer design using Primer 3 (v.0.4.0) [17]. RT-PCR was performed using miRNA samples at different life stages following the method already described. The stability of candidate reference genes was evaluated using geNorm [18] and NormFinder [19]. GeNorm is a widely used algorithm for selection of the most stable reference genes. M values calculated by geNorm represent the stability of each gene with smallest values indicative of highest expression stability. NormFinder is another acknowledged algorithm used for determining the stability of reference genes with smaller values representing higher expression stability. Fold-change differences during development were calculated based on expression during the first time point (day 4) using the ΔΔCt method[20] and normalized to the expression of U6. One-way ANOVA tests were performed to test for differential expression over time using SAS (SAS Statistical Institute, Cary, NC). For genes where expression was significantly different over time, pair wise differences between time points was assessed with Tukey's HSD tests. Type I error was set at alpha = 0.05.

## Results and Discussion

### *In silico* prediction of *D. pulex* miRNAs

From miRBase 16.0, 16,564 mature animal miRNA sequences were obtained to query against the *D. pulex* genome. Following our criteria described above, 1,171 candidate miRNA loci were identified in the *D. pulex* genome. After the secondary structure analysis by mFold, a total of 75 *D. pulex* miRNAs were predicted (Table S1). Two clustered groups of miRNAs, mir-12/mir-283 and mir-100/mir-125, were identified.

### Choosing a stable *D. pulex* reference gene

Several widely used miRNA reference genes (U6 Spliceosomal RNA, RNU1A, RNU5A, SNORD25, and SCARNA17) were selected as candidate reference genes in our study. Out of these, only RNU1A and U6 had BLAST hits to the *D. pulex* genome. After several attempts to amplify RNU1A and U6 using sequence specific primers, only the U6 gene could be successfully amplified. This is not surprising since U6 is one of the most highly conserved spliceosomal RNAs [21]. Next, geNorm and NormFinder were used to test the expression stability of U6 during *D. pulex* development. U6 gene had the smallest M value (0.09) and the smallest stability value (0.15) (Table 1) further supporting its use as a stable reference gene for miRNA studies in *D. pulex* (genes with M values≤1.5 are considered stably expressed).

**Table 1:** M value and stability value of candidate reference genes calculated by geNorm and NormFinder.

miRNA	Stability value	M value
mir8	0.40	0.36
mir9	0.20	0.17
mir12	0.41	0.12
mir92	0.24	0.14
mir100	0.52	0.13
mir153	0.35	0.18
mir283	0.30	0.38
U6	0.15	0.09

Genes with M value≤1.5 are considered stably expressed genes. Stability values represent the combination of intra- and intergroup expression variation.

### *In vivo* validation of *D. pulex* miRNAs

Out of the 14 miRNAs selected for validation, 8 (mir-8, mir-9, mir-12, mir-92, mir-100, mir-133, mir-153 and mir-283) were successfully amplified. This suggests that our computational prediction method is an efficient way to discover conserved miRNAs in *D. pulex*. Since only one pair of primers was tested for each miRNA, additional primers might need to be designed for further validation of the remaining 6 miRNAs (mir-1, mir-10, mir-34, mir-96, mir-124 and mir-137). Because mir-133 expression could only be detected at day 12, it was excluded from the expression stability test and expression changes test.

**Figure 1:**
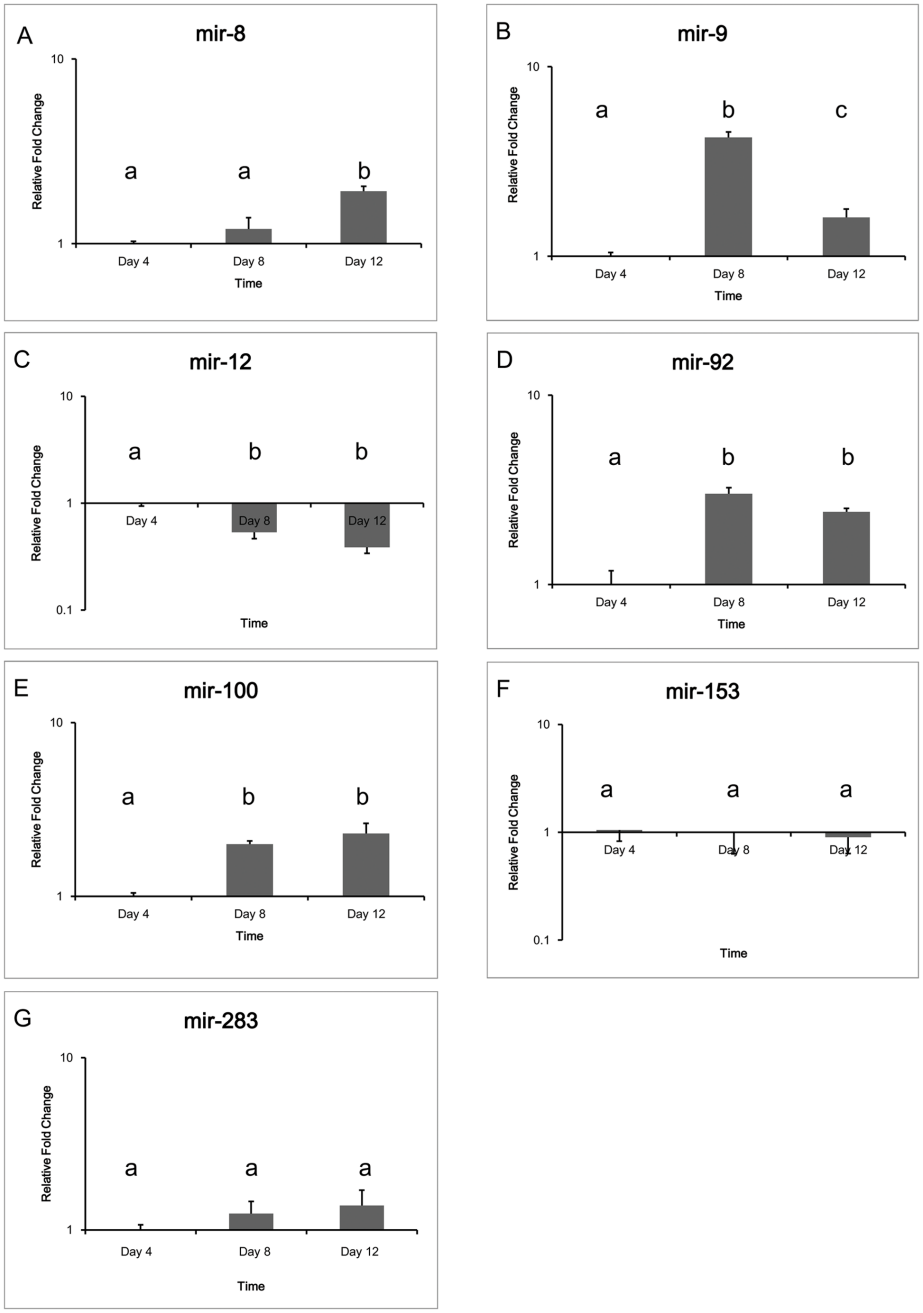
miRNA (mir-8 (A), mir-9(B), mir-12(C), mir-92(D), mir-100(E), mir-153(F), mir283(G)) expression changes during *D. pulex* development. Bars plotted represent means and standard errors. Letters indicate Tukey's groupings (P<0.05) of gene expression level at three different life stages.

With exception of only two miRNAs (mir-153 and mir-283) all miRNAs changed in expression during the three time points monitored, with only one miRNA (mir-12) decreasing in expression at days 8 and 12 (Figure 1). The significant up-regulation of mir-8 at day 12 and of mir-9 at day 8 coincides with dramatic morphological changes including molting, somatic growth, brood chamber development and egg development in *D*. *pulex* [4], [5]. mir-8 is an important insulin signaling regulator that controls body size in *Drosophila* by suppressing its target gene (u-shaped, *ush*) [10], [22] (see Table S3). mir-9 is known to control the timing of neurogenesis [23], [24]. Thus, we propose that mir-8 and mir-9 might also play important roles in *D. pulex* somatic growth and neurogenesis, but further studies are needed to corroborate these findings. mir-12 is known to regulates the MCT1 and MCM6 genes in Wolbachia-infected mosquito cell line[25]. mir-92 is a novel marker for acute leukemia known to increase the proliferation of myeloid cells [26], [27]. mir-100 acts as a tumor suppressor in acute myeloid leukemia by regulating cell differentiation and survival [28], [29]. The roles of mir-92 and mir-100 in *D. pulex* development are unknown at this time. Interestingly, mir-12 and mir-283 are located within a 1 kb region and transcribe the same pri-miRNA, but had opposite expression patterns. This uncoordinated expression profile has also been identified in *Drosophila* [30] and provides evidence of post-translational regulation in these clustered miRNAs.

## Conclusions

In this research, we predicted 75 conserved *D. pulex* miRNAs and successfully validated 8 miRNAs by RT-PCR. Using U6 as reference gene, we tested the expression of these miRNAs during different *D. pulex* life stages (days 4, 8, and 12). Significant changes in the expression of mir-8, mir-9, mir-12, mir-92 and mir-100 were observed, suggesting they play an important role during *Daphnia* development. As a next step, a specific designed *D. pulex* miRNA target prediction program will be developed to better understand the roles that miRNAs play in *D. pulex* development. This study is the first to report expression of miRNAs on *D. pulex* and will facilitate future epigenetic research on this species and daphnids in general.

## Acknowledgments

The authors acknowledge Dr. John Colbourne's lab at Indiana University for providing *D. pulex* and Ashley Chin-Baarstad and Dr. Matthew Hale for their valuable comments.

## Supporting Information

Table S1 **Predicted** ***D. pulex*** **miRNAs, genomic coordinates, and mature miRNA sequences.**
(DOCX)

Table S2 **miRNAs and primer sequences.**
(DOCX)

Table S3 **Biological functions associated with the differentially expressed miRNAs during** ***D. pulex*** **development.**
(DOCX)

File S1 **PERL code for pre-miRNA sequence analyze.**
(TXT)
