# Supplementary material for: In Silico Prediction and In Vivo Validation of Daphnia pulex Micrornas
Source: PLoS One. 2014 Jan 6;9(1):e83708. doi: 10.1371/journal.pone.0083708 (PMC3882220; doi:10.1371/journal.pone.0083708)
Supplement: Table S1 — Predicted D. pulex miRNAs, genomic coordinates, and mature miRNA sequences. (DOCX) [file pone.0083708.s001.docx]

**Table S1. Predicted *D. pulex* miRNAs, genomic coordinates, and mature miRNA sequences.**

| **Predicted miRNA** | **Genomic Coordinates** | **Mature miRNA sequence** |
| --- | --- | --- |
| dpu-mir-1 | scaffold_1:1720944-1720923 | CCGUGCUUCCUUACUUCCCAUA |
| dpu-miR-100 | scaffold_71:446720-446698 | AACCCGUAGAUCCGAACUUGUGU |
| dpu-mir-10 | scaffold_7:304885-304864 | UACCCUGUAGAUCCGAAUUUGU |
| dpu-mir-12 | scaffold_1:1847905-1847883 | UGAGUAUUACAUCAGGUACUGGU |
| dpu-mir-124 | scaffold_120:76940-76962 | UAAGGCACGCGGUGAAUGCCAAG |
| dpu-mir-125 | scaffold_71:445406-445427 | UCACAAGUUAGGGUCUCAGGGA |
| dpu-mir-133 | scaffold_1:1708478-1708457 | UUGGUCCCCUUCAACCAGCUGU |
| dpu-mir-1277a | scaffold_32:794746-794767 | AUAUAUAUAUAUAUAUAUGUAC |
| dpu-mir-1277b | scaffold_100:300960-300981 | CUUUAUAUAUAUAUAUAUGUAC |
| dpu-mir-1322 | scaffold_42:141666-141687 | ACAGCAACAACAGCAGCAGCAA |
| dpu-mir-137 | scaffold_92:410975-410996 | UAUUGCUUGAGAAUACACGUUG |
| dpu-mir-15 | scaffold_28:901796-901817 | CAGCAGCAGCACACAAAACUAU |
| dpu-mir-1502 | scaffold_5030:534-552 | AGACUUUUCAGGUAGUUCG |
| dpu-mir-153 | scaffold_3:3560667-3560646 | UUGCAUAGUCACAAAAGUGAUG |
| dpu-mir-1587a | scaffold_46:689049-689070 | GGGCUGGGCUGGGCUGGGCUGG |
| dpu-mir-1587b | scaffold_200:123544-123565 | GGGCUGGGCUGGGCUGGGCUGG |
| dpu-mir-1814 | scaffold_40:1045656-1045677 | GGUUUUUUGGUUUUGUUUCCUU |
| dpu-mir-184 | scaffold_24:317115-317138 | ACUGGACGGAGAACUGAUAAGGGC |
| dpu-mir-190 | scaffold_3:1681569-1681594 | AGAUAUGUUUGAUAUUCUUGGUUGUU |
| dpu-mir-193 | scaffold_167:85478-85458 | UACUGGCCUGCUAAGUCCCAA |
| dpu-mir-210-3p | scaffold_51:480364-480342 | CUUGUGCGUGUGACAGCGGCUAU |
| dpu-mir-210-5p | scaffold_51:480344-480365 | AGCCGCUGUCACACGCACAAGA |
| dpu-mir-219 | scaffold_253:93636-93657 | AGAAUUGCGUUUGGACAAUCAG |
| dpu-mir-2325 | scaffold_41:201387-201408 | GUUGUUUUUUUUCUUUUUUUUA |
| dpu-mir-263a | scaffold_87:475620-475643 | AAUGGCACUGGAAGAAUUCACGGG |
| dpu-mir-263b | scaffold_87:475817-475840 | CUUGGCACUGGAAGAAUUCACAGA |
| dpu-mir-275 | scaffold_4:1790782-1790804 | UCAGGUACCUGAAGUAGCGCGCG |
| dpu-mir-276 | scaffold_15:755667-755688 | UAGGAACUUCAUACCGUGCUCU |
| dpu-mir-279 | scaffold_63:523674-523653 | UGACUAGAUCCACACUCAUCCA |
| dpu-mir-279a | scaffold_43:177151-177172 | UGACUAGAUCCAUACUCACCAG |
| dpu-mir-279b | scaffold_43:177546-177567 | UGACUAGAUCCAUACUCAUCUA |
| dpu-mir-282 | scaffold_57:216687-216666 | UAGCCUCUCCUAGGCUUUGUCU |
| dpu-mir-283 | scaffold_1:1848761-1848742 | AAAUAUCAGCAGGUAAUUCU |
| dpu-mir-2a | scaffold_80:240622-240644 | UAUCACAGCCAGCUUUGACGAGC |
| dpu-mir-2b | scaffold_80:241083-241106 | UAUCACAGCCAGCUUUGAUGAGCG |
| dpu-mir-305 | scaffold_4:1791164-1791187 | AUUGUACUUCAUCAGGUGCUCUGG |
| dpu-mir-307-3p | scaffold_47:641122-641102 | UCACAACCUCCUUGAGUGAGU |
| dpu-mir-307-5p | scaffold_47:641084-641103 | UCACAACCUCCUUGAGUGAG |
| dpu-mir-309 | scaffold_24:361445-361466 | AGUCACUGGGUAAAGUUUGUCC |
| dpu-mir-313 | scaffold_32:710981-711002 | GAAUGUUGUGAAUAGUGUAAUA |
| dpu-mir-315 | scaffold_58:431908-431930 | UUUUGAUUGUUGCUCAGAAAGCC |
| dpu-mir-317 | scaffold_4:1243985-1243961 | UGAACACAGCUGGUGGUAUCUCAGU |
| dpu-mir-34 | scaffold_4:1242114-1242091 | UGGCAGUGUGGUUAGCUGGUUGUG |
| dpu-mir-341 | scaffold_53:695412-695431 | UCGGUCGAUCGGUCGGUGGU |
| dpu-mir-375 | scaffold_55:302090-302111 | UUGUUCGUUUGGCUCGAGCAGC |
| dpu-mir-4507 | scaffold_200:122781-122802 | CUGGGUUGGGCUGGGCUGAAUA |
| dpu-mir-466a | scaffold_9:248987-249008 | AGAGACACACACACACACAAGU |
| dpu-mir-466b | scaffold_19:1276861-1276882 | GUGUGUGUGUGUGAGUGUGUAC |
| dpu-mir-466c | scaffold_30:678246-678266 | UGUGUGUGUGUAUGUGUGUAA |
| dpu-mir-466d | scaffold_67:693500-693521 | GUGUGUGUGUGAGUGUGUGUGU |
| dpu-mir-466e | scaffold_169:10076-10095 | UUGUGUGUGUGAGUGUGUGU |
| dpu-mir-466f | scaffold_6332:737-758 | GUGUGUGUGUGAGUGUGUGAUU |
| dpu-mir-467 | scaffold_397:474-495 | UACAUACACACACAUAUACUAU |
| dpu-mir-5106 | scaffold_76:500686-500706 | GUCGGUAGCUCAGUUGGCAGA |
| dpu-mir-513 | scaffold_54:604492-604513 | ACAGGGAGGUGUCAUUUACUAC |
| dpu-mir-541 | scaffold_3:2756631-2756652 | GGCGAACACAGAAUCCAUUCUU |
| dpu-mir-5416 | scaffold_12:1407447-1407468 | AUUCCUUUUCUCUUUUUUCUUU |
| dpu-mir-574a | scaffold_5:670353-670374 | GUGUGUGUGUGUGAGUGUCGGA |
| dpu-mir-574b | scaffold_5:1791308-1791288 | GUGUGUGUGUGUGAGUGGUGU |
| dpu-mir-638 | scaffold_7427:1389-1410 | AGGGAUCGCGGGCGGGCGGCCC |
| dpu-mir-669 | scaffold_88:40467-40488 | UACACACACACACACAAGUAUU |
| dpu-mir-71 | scaffold_80:240430-240452 | UGAAAGACAUGGGUAGUGAGAUG |
| dpu-mir-79 | scaffold_2:1526251-1526273 | AUAAAGCUAGGUUACCAAAGUUA |
| dpu-mir-7a | scaffold_191:112613-112589 | UGGAAGACUAGUGAUUUUGUUGUUC |
| dpu-mir-7b | scaffold_11571:1094-1070 | UGGAAGACUAGUGAUUUUGUUGUUC |
| dpu-mir-8 | scaffold_131:139446-139468 | UAAUACUGUCAGGUAAAGAUGUC |
| dpu-mir-87 | scaffold_1:2191121-2191142 | GUGAGCAAAGUUUCAGGUGCGU |
| dpu-mir-9 | scaffold_2:1526213-1526235 | UCUUUGGUUAUCUAGCUGUAUGA |
| dpu-mir-92 | scaffold_38:876194-876215 | UAUUGCACUCGUCCCGGCCUGU |
| dpu-mir-967 | scaffold_1088:1977-1957 | CUUUUCCACCUAGGUGUUUCU |
| dpu-mir-981 | scaffold_2:1451012-1450991 | UUCGUUGUCGACGAAACCUGCA |
| dpu-mir-993 | scaffold_7:282360-282382 | GAAGCUCGUUUCUACAGGUAUCU |
| dpu-mir-bantam | scaffold_115:370209-370231 | UGAGAUCAUUGUGAAAGCUGAUU |
| dpu-mir-iab-4-3p | scaffold_7:515582-515605 | CGGUAUACCUUCAGUAUACGUAAC |
| dpu-mir-iab-4-5p | scaffold_7:515547-515568 | ACGUAUACUGAAUGUAUCCUGA |
