# Supplementary material for: In Silico Prediction and In Vivo Validation of Daphnia pulex Micrornas
Source: PLoS One. 2014 Jan 6;9(1):e83708. doi: 10.1371/journal.pone.0083708 (PMC3882220; doi:10.1371/journal.pone.0083708)
Supplement: Table S2 — miRNAs and primer sequences. (DOCX) [file pone.0083708.s002.docx]

**Table S2. miRNAs and primer sequences.**

| mir-8 | Mature miRNA sequence | UAAUACUGUCAGGUAAAGAUGUC |
| --- | --- | --- |
|  | RT primer | GTCGTATCCAGTGCAGGGTCCGAGGTATTCGCACTGGATACGACGACATA |
|  | Forward primer | CGCCGATAATACTGTCAGGTAAA |
| mir-9 | Mature miRNA sequence | UCUUUGGUUAUCUAGCUGUAUGA |
|  | RT primer | GTCGTATCCAGTGCAGGGTCCGAGGTATTCGCACTGGATACGACTCATAC |
|  | Forward primer | AGCGCTCTTTGGTTATCTAGCT |
| mir-10 | Mature miRNA sequence | UACCCUGUAGAUCCGAAUUUGU |
|  | RT primer | GTCGTATCCAGTGCAGGGTCCGAGGTATTCGCACTGGATACGACACAAAT |
|  | Forward primer | CGTGAATGGGACATCTAGGCT |
| mir-12 | Mature miRNA sequence | UGAGUAUUACAUCAGGUACUGGU |
|  | RT primer | GTCGTATCCAGTGCAGGGTCCGAGGTATTCGCACTGGATACGACACCAGT |
|  | Forward primer | ACGGCTGAGTATTACATCAGGT |
| mir-92 | Mature miRNA sequence | UAUUGCACUCGUCCCGGCCUGU |
|  | RT primer | GTCGTATCCAGTGCAGGGTCCGAGGTATTCGCACTGGATACGACGCAGGC |
|  | Forward primer | GGATCAATTGCACTCGTCCCG |
| mir-100 | Mature miRNA sequence | AACCCGUAGAUCCGAACUUGUGU |
|  | RT primer | GTCGTATCCAGTGCAGGGTCCGAGGTATTCGCACTGGATACGACACACAA |
|  | Forward primer | TGACCAACCCGTAGATCCGAAC |
| mir-133 | Mature miRNA sequence | UUGGUCCCCUUCAACCAGCUGU |
|  | RT primer | GTCGTATCCAGTGCAGGGTCCGAGGTATTCGCACTGGATACGACACAGCT |
|  | Forward primer | GCATGAACCAGGGGAAGTTGG |
| mir153 | Mature miRNA sequence | UUGCAUAGUCACAAAAGUGAUG |
|  | RT primer | GTCGTATCCAGTGCAGGGTCCGAGGTATTCGCACTGGATACGACCATCAC |
|  | Forward primer | CGCGATTGCATAGTCACAAAA |
| mir-283 | Mature miRNAsequence | AAAUAUCAGCAGGUAAUUCU |
|  | RT primer | GTCGTATCCAGTGCAGGGTCCGAGGTATTCGCACTGGATACGACAGAATT |
|  | Forward primer | GCGCGAAATATCAGCAGGT |
| Universal | Reverse primer | GTGCAGGGTCCGAGGT |
| U6 | Forward primer | CGATACAGAGAAGATTAGCATGG |
|  | Reverse primer | TTCACGATTTTGCGTGTCAT |
