# Supplementary material for: In Silico Prediction and In Vivo Validation of Daphnia pulex Micrornas
Source: PLoS One. 2014 Jan 6;9(1):e83708. doi: 10.1371/journal.pone.0083708 (PMC3882220; doi:10.1371/journal.pone.0083708)
Supplement: Table S3 — Biological functions associated with the differentially expressed miRNAs during D. pulex development. (DOCX) [file pone.0083708.s003.docx]

**Table S3. Biological functions associated with the differentially expressed miRNAs during *D. pulex* development.**

| miRNA Name | Function | Reference |
| --- | --- | --- |
| mir-8 | Controls body size  Negative regulator of Wnt signaling | Jin *et al.*(2012) [10]  Kennell *et al.*(2008)[22] |
| mir-9 | Inhibition of cell proliferation in breast cancer cells  Controls timing of neurogenesis | Selcuklu *et al.*(2011)[23]  Coolen *et al.* (2012)[24] |
| mir-12 | Potential regulator of MCT1(monocarboxylate transporter isoform 1) and MCM6 (mini-chromosome maintenance protein 6) in mosquito cell line | Osei-Amo *et al.* (2012)[25] |
| mir-92 | Down-regulation in acute leukemia  Regulation of myeloid cells proliferation | Tanaka *et al.*(2009)[26]  Manni *et al.* (2009)[27] |
| mir-100 | Regulation of cell death and differentiation in acute leukemia  Potential tumor suppressor | Zheng *et al.*(2012)[28]  Giangrece *et al.*(2013)[29] |
